# Effect of Praziquantel on *Schistosoma mekongi* Proteome and Phosphoproteome

**DOI:** 10.3390/pathogens9060417

**Published:** 2020-05-27

**Authors:** Peerut Chienwichai, Sumate Ampawong, Poom Adisakwattana, Tipparat Thiangtrongjit, Yanin Limpanont, Phiraphol Chusongsang, Yupa Chusongsang, Onrapak Reamtong

**Affiliations:** 1 Faculty of Medicine and Public Health, HRH Princess Chulabhorn College of Medical Science, Chulabhorn Royal Academy, Bangkok 10210, Thailand; peerut.chi@pccms.ac.th; 2 Department of Tropical Pathology, Faculty of Tropical Medicine, Mahidol University, Bangkok 10400, Thailand; sumate.aum@mahidol.ac.th; 3 Department of Helminthology, Faculty of Tropical Medicine, Mahidol University, Bangkok 10400, Thailand; poom.adi@mahidol.ac.th; 4 Department of Molecular Tropical Medicine and Genetics, Faculty of Tropical Medicine, Mahidol University, Bangkok 10400, Thailand: tipparat.thi@mahidol.ac.th; 5 Department of Social and Environmental Medicine, Faculty of Tropical Medicine, Mahidol University, Bangkok 10400, Thailand; yanin.lim@mahidol.ac.th (Y.L.); phiraphol.chu@mahidol.ac.th (P.C.); yupa.chu@mahidol.ac.th (Y.C.)

**Keywords:** *Schistosoma mekongi*, praziquantel, proteomics, phosphoproteomics, endoplasmic reticulum-associated degradation, Src kinase

## Abstract

*Schistosoma mekongi* causes schistosomiasis in southeast Asia, against which praziquantel (PZQ) is the only treatment option. PZQ resistance has been reported, thus increasing the requirement to understand mechanism of PZQ. Herein, this study aimed to assess differences in proteome and phosphoproteome of *S. mekongi* after PZQ treatment for elucidating its action. Furthermore, key kinases related to PZQ effects were predicted to identify alternative targets for novel drug development. Proteomes of *S. mekongi* were profiled after PZQ treatment at half maximal inhibitory concentration and compared with untreated worms. A total of 144 proteins were differentially expressed after treatment. In parallel, immunohistochemistry indicated a reduction of phosphorylation, with 43 phosphoproteins showing reduced phosphorylation, as identified by phosphoproteomic approach. Pathway analysis of mass spectrometric data showed that calcium homeostasis, worm antigen, and oxidative stress pathways were influenced by PZQ treatment. Interestingly, two novel mechanisms related to protein folding and proteolysis through endoplasmic reticulum-associated degradation pathways were indicated as a parasiticidal mechanism of PZQ. According to kinase–substrate predictions with bioinformatic tools, Src kinase was highlighted as the major kinase related to the alteration of phosphorylation by PZQ. Interfering with these pathways or applying Src kinase inhibitors could be alternative approaches for further antischistosomal drug development.

## 1. Introduction

Schistosomiasis, also known as bilharzia, is a neglected tropical disease that infects over 200 million people worldwide each year, resulting in 200,000 deaths [1,2]. *Schistosoma mekongi* is the causative agent of intestinal schistosomiasis in the lower Mekong river region, especially in Laos and northern Cambodia [1]. Infections in other parts of the world are occasionally reported owing to human migration and travel-related illness [3,4,5]. Clinical manifestations of Mekong schistosomiasis include abdominal pain, bloody stool, diarrhea and liver enlargement, resulting in fatality in severe cases [1,6].

The only drug currently available for the treatment and prevention of *S. mekongi* is praziquantel (PZQ) [7,8]. PZQ has long been considered the drug of choice for schistosomiasis [7]. It has been extensively used for the treatment and control of blood fluke infections and other parasitic diseases for both medical and veterinary purposes [7,9]. PZQ shows excellent efficacy against various species of worm; however, drug resistance and low susceptibility of parasites have been recently observed [10,11]. PZQ resistance has been reported in various species of worms, including *S. mansoni* [12,13], *S. japonicum* [14], *S. hematobium* [15] and other cestode species [16,17]. For blood flukes, PZQ is effective only on adult and cercarial stage worms, while juvenile stage schistosomulae can retain viability after chemotherapy and progress to adulthood [18,19]. In addition, PZQ shows no protection against re-infection, making disease control difficult in high endemic areas [18]. To overcome these problems, a thorough understanding of the mechanism of action of PZQ is required and novel drug targets should be studied. Many hypotheses have been proposed to explain the helminthotoxic effect of PZQ in several *Schistosoma* species; for example, interference of cellular calcium homeostasis [20,21,22], damaging the tegument and exposure of worm antigen to host immunity [20,22,23] and induction of oxidative stress [22,24]. Alterations in protein phosphorylation may also be one of the effects of PZQ in blood fluke killing; however, only a few studies have focused on this [25]. Differential protein phosphorylation throughout development has been detected using anti-phospho antibodies, suggesting the importance of phosphorylation in *S. mansoni* [26]. Moreover, sub-lethal dose of PZQ induced over-expression of Ca^2+^/calmodulin-dependent protein kinase II (CamKII), key kinase for calcium homeostasis, indicating the possible link between the drug and phosphorylation [27]. Information on the effect of PZQ on protein phosphorylation in *Schistosoma* parasites would fill the knowledge void on the mechanism of action of this drug as well as providing some clues for future drug development targeting protein phosphorylation.

Phosphorylation is an important modification of proteins that play fundamental roles in the regulation of cellular metabolism, growth and division. Changes in phosphorylation levels can critically impact the functions proteins thereby reducing the survivability of organisms. Modern mass spectrometric (MS) technologies and phosphoprotein enrichment methods have been successfully used to elucidate the mechanism of action of a number of drugs, such as gemcitabine [28], midostaurin [29] and pyrazolo [3,4-d] pyrimidine [30]. The aim of this study was to apply proteomic and phosphoproteomic approaches to understand the mechanism of action of PZQ in *S. mekongi*. Moreover, the integration of phosphoproteomic and bioinformatics data can be used to predict the main kinases that control and alter of the phosphoproteome. The findings of this study could lead to a better understanding of the molecular action of PZQ in schistosome. In addition, the key kinases affected by PZQ exposure may be potential targets for antischistosomal drug development.

## 2. Results

### 2.1. Anthelmintic Assay of PZQ on S. Mekongi

**Figure 1:**
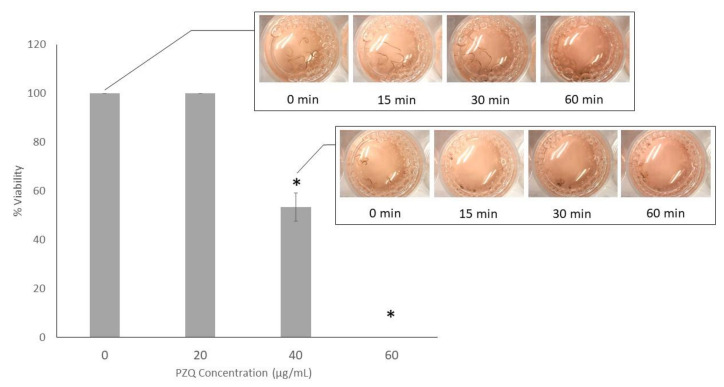
The 40 μg/mL concentration of praziquantel (PZQ) reduced viability of the worms by approximately 46.7%. *S. mekongi* were treated with increasing concentration of PZQ for an hour and parasites without movement for a minute were assumed as dead. Bar chart represents percentage of viable *S. mekongi* after PZQ treatment. Asterisk shows statistical significance (*p* value < 0.05).

To examine the effective dose of PZQ for parasiticide activity on *S. mekongi*, adult worms were exposed to various concentrations of PZQ for 60 min and worm viability was assessed by observing the movement of the worms under a video microscope. PZQ at 0 and 20 μg/mL had no effect with all worms moving periodically (Video S1). After 40 μg/mL-PZQ treatment, only half of the worms moved, indicating some parasiticidal activity (Figure 1, Video S2). At PZQ doses higher than 40 μg/mL, all worms were killed (no movement observed). A PZQ concentration of 40 μg/mL killed 46.7% of adult worms and was defined as IC_50_. Worms treated with 40 μg/mL-PZQ were further investigated for protein expression alteration.

### 2.2. Effects of PZQ on S. Mekongi Proteome

**Figure 2:**
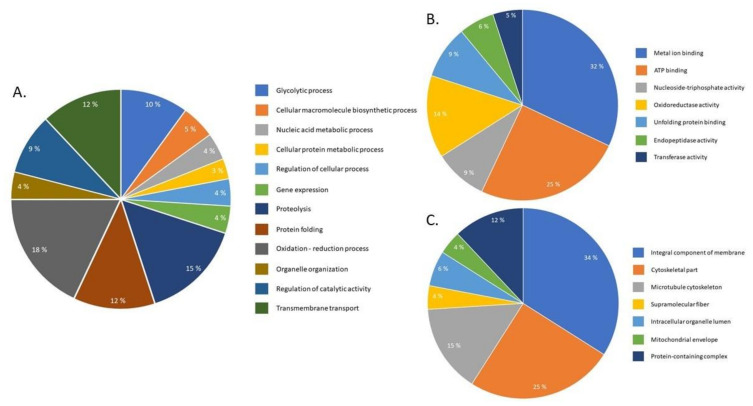
Gene ontology classification of *S. mekongi* proteomic data after PZQ treatment. The Differential proteins after PZQ exposure were classified according to their gene ontology using Blast2Go. Three terms of gene ontologies including (**A**) biologic process, (**B**) molecular function, and (**C**) cellular component are presented.

The proteomic approach identified 1076 *S. mekongi* proteins in which 144 proteins were differentially expressed after PZQ treatment. The abundance of 68 proteins increased (Table S1), while 76 proteins decreased (Table S2). Gene ontology analysis of differential proteins was performed using BLAST2GO bioinformatic software. According to the biologic process term, most differentially expressed proteins are involved in redox processes (18%) and proteolysis (15%) (Figure 2A). However, using a molecular function term indicated that the proteins mainly correlated to metal-ion (32%) and ATP binding (25%) (Figure 2B). In the cellular component term, most of the altered proteins related to integral membrane (34%) and cytoskeletal components (25%) (Figure 2C). The top twenty up- and downregulated *S. mekongi* proteins after PZQ treatment are shown in Table 1 and Table 2, respectively. Proteins related to structure, energy, protease, antioxidant, transcription and translation and antigens were highly upregulated after PZQ exposure. The structural protein paramyosin was the most upregulated protein with 5.96-fold change after treatment. However, some proteins involved in structure, energy, stress response and signaling were highly downregulated. The energy metabolism protein aldehyde dehydrogenase 1B1 precursor was the most downregulated protein after PZQ exposure (4-fold decrease). Interestingly, while none of kinases were found to be highly upregulated, there was a significant number of kinases showing decreased expression after PZQ treatment, such as pyruvate kinase PKM (A0A095B084: 2.38-fold decrease), arginine kinase (C1LFZ8: 2.27-fold decrease) and inositol hexakisphosphate and diphosphoinositol–pentakisphosphate kinase 2 (A0A095ASB2: 2-fold decrease) (Table 2).

### 2.3. Effects of PZQ on S. Mekongi Phosphorylation and Phosphoprotein Abundance

**Figure 3:**
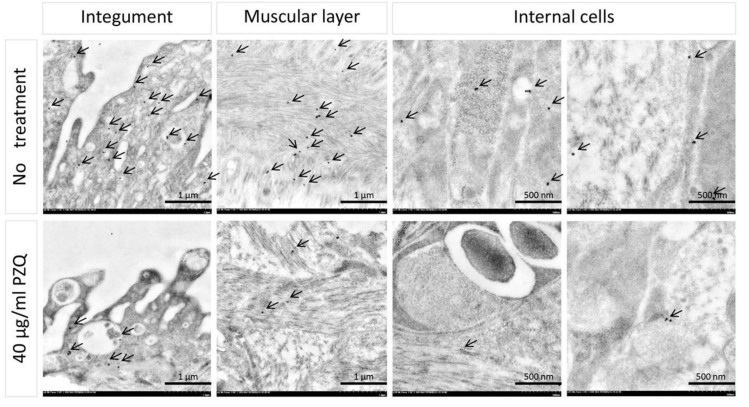
Level of phosphorylation in tegument, muscle layer and internal cells decreased after PZQ treatment. Phosphorylation on untreated and PZQ-treated *S. mekongi* were tagged by anti-phosphoserine antibody. Immune-gold labeling and electron microscope were used to visualize parasite phosphorylation. Phosphorylation is indicated by black spots with arrows. The numbers of spots are markedly reduced in all organs after PZQ treatment.

Levels of phosphorylation in PZQ-treated *S. mekongi* was examined by immunogold labeling and visualized by transmission electron microscope (TEM) and compared with untreated parasites. Anti-phosphoserine was used to determine phosphorylation levels and tissue localization in the parasites. In Schistosoma parasite, the main phosphorylated residues are serine (67.8%), threonine (20.1%) and tyrosine (12.1%) [31]. Phosphorylation of tegument, muscle and internal cells of the worms were markedly reduced after PZQ treatment, as indicated by the black spots in Figure 3. The reduction of phosphorylation could be one of the effects of PZQ treatment. To explore the mechanism of action of PZQ on parasite phosphorylation, phosphoprotein abundance of untreated and PZQ-treated *S. mekongi* were profiled using MS-based phosphoproteomics.

**Figure 4:**
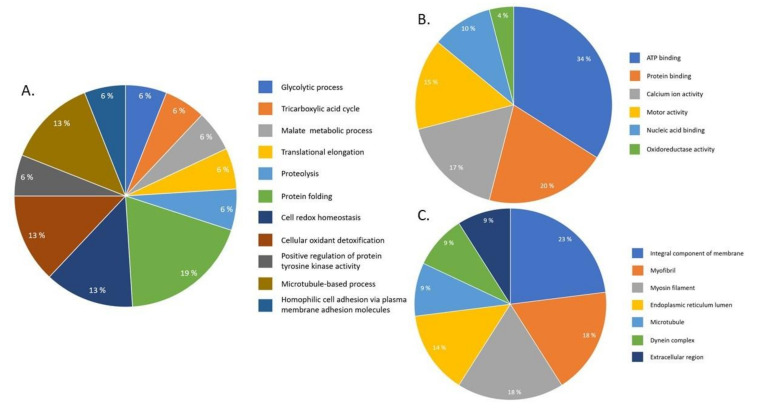
Gene ontology classification of *S. mekongi* phosphoproteomic data after PZQ treatment. The differential phosphoproteins after PZQ exposure were classified according to their gene ontology using Blast2Go. Three terms of gene ontologies including (**A**) biologic process, (**B**) molecular function and (**C**) cellular component are presented.

A total of 448 phosphoproteins of *S. mekongi* were identified using TALON PMAC Magnetic Phospho Enrichment Kit and MS. Among these, 43 phosphoproteins differed after PZQ exposure. Twenty-eight phosphoproteins were upregulated and 15 were downregulated after PZQ treatment. MS-identified phosphopeptides of up- and downregulated phosphoproteins were 124 and 44 peptides, respectively (Table 3 and Table 4). Phosphoproteins involved in structure, energy, protease, antigen, transcription and translation, immune system, stress response, transporters and antioxidants were upregulated. An unknown protein, SJCHGC01909, was the most upregulated phosphoprotein after PZQ treatment (6.13-fold increase). Some phosphoproteins involved in structure, energy, antigen, scaffold, antioxidant, transcription and translation, stress response and signaling were also downregulated. Energy metabolism protein aldolase was the most downregulated phosphoprotein (2.38-fold decrease). Gene ontology classification of differential phosphoproteins after PZQ exposure using the biologic process term showed that differential phosphoproteins mostly affected protein folding (19%), cell redox homeostasis (13%), cellular oxidant detoxification (13%) and microtubule-based process (13%) (Figure 4A). Furthermore, the molecular function term classification revealed that most altered phosphoproteins related to ATP binding (34%) and protein binding (20%) (Figure 4B). For the cellular component term, most differential phosphoproteins were integral membrane (23%), myofibril (18%) and myosin filament (18%) components (Figure 4C).

**Figure 5:**
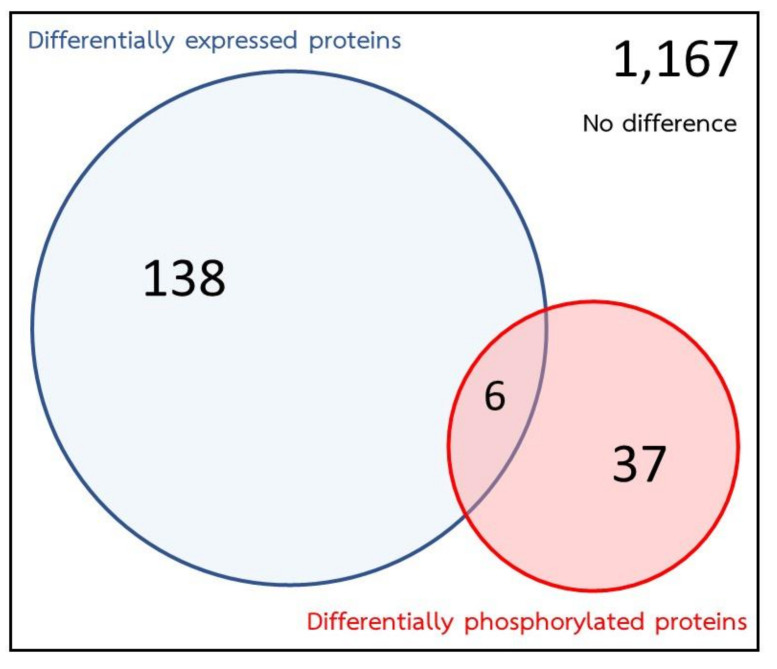
Distribution of proteomic and phosphoproteomic data. Identification of proteins by mass spectrometer showed the total of 1167 proteins. Expression levels of 138 proteins changed after PZQ treatment without altering their phosphorylation. Thirty-seven phosphoproteins changed without changing their expression. Six proteins were altered both of their expression and phosphorylation.

The integrated proteomic and phosphoproteomic data revealed that 1348 proteins could be identified. After *S. mekongi* were exposed to PZQ, a total of 144 proteins were identified by proteomics as showing differential expression and 43 phosphoproteins were altered as identified by phosphoproteomics. However, 138 proteins were found to be altered by proteomics analysis alone. These proteins may indicate the effect of PZQ at the protein expression level. Likewise, 37 proteins were found to be altered by phosphoproteomic analysis alone, which could reflect the effect of PZQ at the phosphorylation level. Only six proteins—paramyosin, cathepsin B, annexin, glutathione S-transferase, aldolase and heat shock protein—showed the alterations by both proteomic and phosphoproteomic approaches (Figure 5).

### 2.4. Pathway Analysis

**Figure 6:**
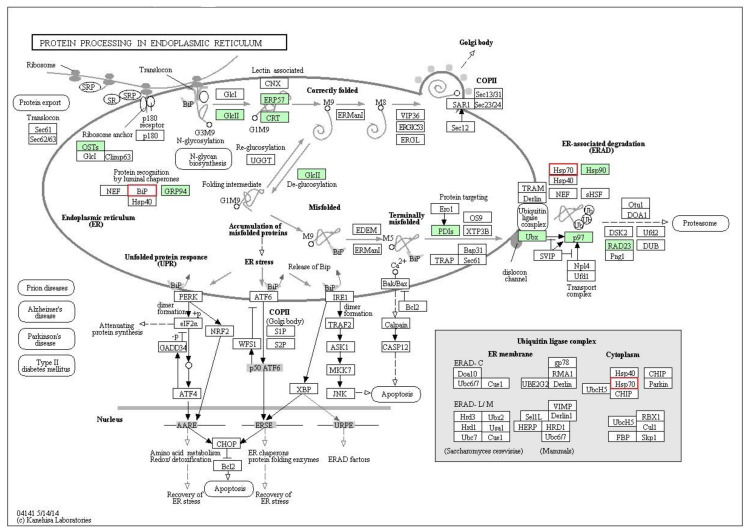
Differentially expressed and phosphorylated proteins after PZQ treatment by the protein processing endoplasmic reticulum (ER) pathway. Differential proteins and phosphoproteins were analyzed for the potential pathways which were affected by PZQ using KEGG Automatic Annotation Server (KAAS) bioinformatic tool. Protein processing ER pathway was highlighted as the major pathway. The differential proteins and phosphorylated are indicated by green boxes and red borders, respectively.

To fully understand the anthelmintic mechanisms of PZQ, the integral proteomic and phosphoproteomic data were further investigated by KAAS bioinformatic software. The result suggested that protein processing in the endoplasmic reticulum (ER) (pathway ID: 04,141) was the main enriched pathway in this study. As shown in Figure 6, differentiations of 10 proteins (green boxes) and two phosphoproteins (red borders) were detected in this pathway after treatment. Furthermore, most differential proteins and phosphoproteins affected by PZQ were mapped to protein misfolding and ER-associated degradation (ERAD). These two processes control the quality of newly synthesized proteins and degrade misfolded proteins. Findings of KAAS analysis correlated with the gene ontology classification, which demonstrated that protein folding, unfolding protein binding, proteolysis and endopeptidase activity were altered by PZQ treatment (Figure 2 and Figure 4). Therefore, alteration of protein processing in the ER, especially on the ERAD pathway, may be a possible mechanism of action of PZQ in *S. mekongi*. The integrated proteomic and phosphoproteomic data not only provided a novel PZQ mechanism, but also supported the putative PZQ mechanisms on schistosome including calcium homeostasis, tegument damage and induction of oxidative stress. There were 9, 10 and 28 proteins with altered regulation in calcium homeostasis, worm antigens and oxidative stress, respectively (Table 5).

### 2.5. Prediction of Key Kinases Effected by PZQ

**Figure 7:**
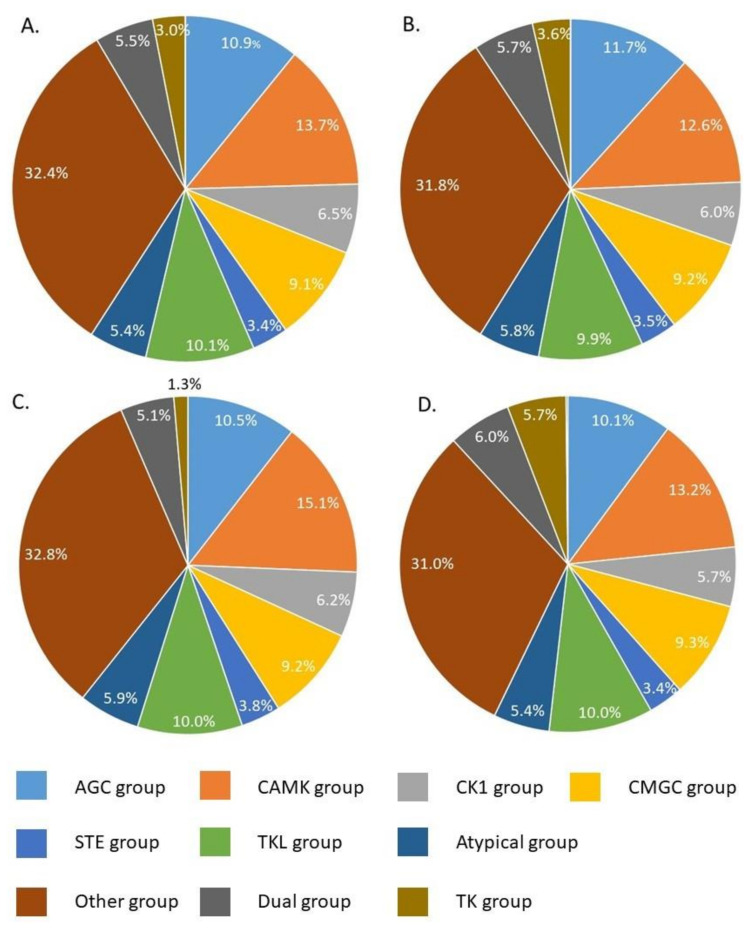
Distribution of kinase groups involved in protein phosphorylation in vital pathways of *S. mekongi.* Phosphopeptides identified from differential phosphoproteins were used to predict kinases corresponding for their phosphorylation with Group-based Prediction System. Percentage of phosphorylation sites for predicted kinases in (**A**) calcium homeostasis, (**B**) worm antigen, (**C**) oxidative stress and (**D**) proteins folding and proteolysis are shown.

Phosphorylation of proteins in *S. mekongi* was strongly affected by PZQ, as shown by immunohistochemistry and phosphoproteomic analyses. Kinases are the key enzymes in the control of phosphorylation levels in cells and are known to be potential drug targets in several diseases. Using phosphopeptides and phosphorylation sites obtained from the MS results, it was possible to predict kinase–substrate interactions by the group-based prediction system. Phosphorylation data of calcium homeostasis, worm antigen, oxidative stress, protein folding, and proteolysis were subjected to kinase–substrate prediction to identify key kinases. Results showed 83 kinases (11 kinase groups) were involved in phosphorylation of proteins in these four pathways (Figure 7). Among the 83 kinases, proto-oncogene tyrosine-protein kinase Src, calcium/calmodulin-dependent protein kinase-like (CAMKL) and Wee protein kinase were the top three kinases controlling the pathways of interest (Table S3). Inhibition of these kinases would interfere with phosphorylation and signal transduction of calcium homeostasis, worm antigens, oxidative stress, protein folding and proteolysis pathways in a manner comparable to PZQ. Applying inhibitors of these kinases could therefore be an alternative means of schistosomiasis treatment.

## 3. Discussion

*S. mekongi* is a public health threat in South–East Asia with PZQ currently the only drug of choice for treatment of this blood fluke. This study aimed to understand the parasiticidal mechanisms of PZQ on *S. mekongi* using proteomic and phosphoproteomic approaches. To study the parasiticidal effect of PZQ, IC_50_ was established by administering the drug to worms and observing for their movement. PZQ at 40 μg/mL killed 46.7% of adult worms and was defined as IC_50_ (Figure 1). In previous studies, the IC_50_ of PZQ varied from 0.03 to 80 μg/mL [29,32,33,34]. Duration of exposure could have an effect on IC_50_. Our study exposed adult worms to PZQ for 60 min before observing the mortality rates. This correlates with data indicating that PZQ starts to affect worms a few minutes after exposure [20]. The 60 min incubation period in this study could have allowed more time for the drug to penetrate into the worms to disrupt their protein moieties.

Several studies have proposed mechanisms of action of PZQ on schistosome. However, the exact molecular mechanism of PZQ against *Schistosoma spp.* is still unclear. High-throughput technologies were applied to *Schistosoma* parasite with reduced susceptibility to PZQ for elucidating its mechanism of action. Proteomic analysis of less-susceptible *S. mansoni* to PZQ revealed the upregulation of 2 proteins; heat shock protein 70 and calcium ATPase 2 protein [35]. Our study found altered expression of the same protein or proteins with similar function; heat shock protein 70 (O45039) and sodium/potassium-transporting ATPase subunit alpha (A0A095AFH4, A0A094ZR23) (Table S1,2). Furthermore, gene expression profiling of less-susceptible parasite indicated a list of gene involving with calcium signaling pathway (calcium binding protein, calmodulin, calponin) and oxidative stress response (thioredoxin and superoxide dismutase) [36]. Our mass spectrometric data also found proteins in those pathways, which were 20 kDa calcium-binding protein (A0A095A3D3), putative annexin (G4VL68), voltage-dependent anion-selective channel protein (VDAC) (G4M1U8) for calcium signaling pathway and tryparedoxin peroxidase (C1LV40), glutathione-S-transferase (Q26513) for oxidative stress pathway (Table 5). The mechanisms of action of PZQ have been continuously investigated. The interference of the calcium signaling pathway is one of the putative PZQ mechanisms of schistosome [7,20,21,37,38]. Pharmacologically, PZQ induces calcium influx to worm myocytes and contraction of musculature, leading to paralysis and loss of worm movement [20,21]. Voltage-gated calcium channels have been proposed as potential targets for PZQ by interfering with the interaction of the α and β subunit of the proteins, thereby causing excessive uptake of calcium into cells [21,39]. Our MS results revealed the alteration of proteins involving in calcium signaling and interaction, such as the 20 kDa calcium-binding protein (A0A095A3D3), putative annexin (G4VL68) and voltage-dependent anion-selective channel protein (VDAC) (G4M1U8) (Table 5). VDAC controls calcium movement between the mitochondria and cytoplasm; inhibition of this protein causes apoptosis and has been used as a drug target for cancer treatment [40,41]. Therefore, alteration of calcium homeostasis is strongly suggested as one of the parasiticidal mechanisms of PZQ. Another parasiticidal mechanism of PZQ against schistosome is the increased susceptibility of the parasite to host immunity by exposing antigens on worm surface [20]. Antibody and cell-mediated immunity significantly impact the killing ability of PZQ [20,23]. PZQ was less effective against *S. mansoni* in T cell-deprived hosts than normal hosts [42]. Immunization of mice with worm antigens enhanced the worm-killing effect of PZQ [43]. The studies stated that the worm antigens and host immunity play important roles in the parasiticidal ability of PZQ on schistosome, although the detailed mechanism is still unknown. Our findings found that expression of several antigenic proteins increased after PZQ treatment, including 55 kDa antigen myophilin (Q86DV9) (Table 5), which may play some role in the effects of PZQ. Oxidative stress induction was also proposed as a mechanism of PZQ to parasitic worms [24,44]. The pathway for PZQ to induce oxidative stress in parasite is still unknown, although schistosome and other parasitic flatworms increased expression of antioxidants in response to PZQ [24,37,44,45]. Our study indicated that PZQ treatment increased expression of antioxidant proteins, such as tryparedoxin peroxidase (C1LV40) and glutathione-S-transferase (Q26513). In addition, phosphorylation of thioredoxin peroxidase (O97161), 26 kDa glutathione-S-transferase (G4LXF8) and peroxiredoxin-2 (A0A095A0V3) were altered (Table 5). Our findings also revealed a potential novel mechanism of PZQ: the alteration of protein folding and proteolysis through the ERAD pathway. The function of the ERAD pathway is to eliminate unfolded/misfolded proteins from the ER through a series of proteins and phosphoproteins [46]. ERAD tags unfolded/misfolded proteins with ubiquitin prior to sending them for proteasome-mediated degradation [47]. Precise activity of ERAD is crucial for quality control of structure and function of the protein repertoire [48]. Interruption of ERAD could cause the accumulation of toxic proteins, apoptosis and cell death, which has been implicated in many diseases, such as Alzheimer’s disease, Parkinson’s disease and rheumatoid arthritis [47,48]. In *Trypanosoma brucei*, ERAD plays important roles in the processing of antigenic variation for host immune evasion [49]. *T. brucei* is believed to survive in extreme stress conditions, such as blood, by using unique ERAD mechanisms to shift their surface antigens and avoid detection by the immune system [46,49]. Moreover, *Plasmodium falciparum* showed sensitivity to ERAD inhibitor treatment. Therefore, proteins in ERAD may be novel targets for malarial drug development [50,51]. Unlike blood-dwelling protozoa, the process of antigenic variation of schistosome is manipulated by alternative splicing of micro-exon genes [52,53]. Although the schistosome ERAD pathway has never been studied before, protein break down and degradation was proposed as drug target for schistosomiasis treatment in a previous study [54]. Here, we propose that PZQ exerts its effect by interfering with the ERAD pathway of *S. mekongi.*

Phosphorylation is a vital molecular process for blood fluke biology, the alteration of which affects the viability of the worms. Our immunogold labeling to phosphoserine showed an overall reduction of phosphorylation levels after PZQ treatment (Figure 3). This present study found changes of a number of phosphoproteins after PZQ treatment. However, none of target validation was performed which is the limitation of this study. In addition, the inhibition of specific kinases, protein kinase C (PKC) and extracellular signal-regulated kinases, resulted in a reduction of protein phosphorylation and decreases in attachment, muscular activity, neuromuscular coordination, pairing and reproductive function of schistosomes [26]. Recently, drugs acting on phosphorylation and protein kinases have been proposed as novel targets for schistosomiasis treatment [25,26,55,56,57]. Kinomic array of *S. mansoni* revealed a number of kinases those are vital for *Schistosoma* biology [31] in which Src kinase, Cyclin-dependent kinase (CDK), PKC were predicted from bioinformatic tool as the critical kinases for anthelminthic effects of PZQ (Table S3). Our study indicates that kinases of critical pathways in the parasite and Src kinase are important kinases for PZQ response (Figure S1, Table S3). Src kinase is a cytoplasmic tyrosine kinase that controls diverse cellular processes, including adhesion, proliferation and differentiation [25]. Only a few studies have focused on the inhibition of Src kinase in schistosomes. An inhibitor of Src kinase, Herbimycin A, suppresses the mitotic activity and egg production of female schistosome, resulting in reduced reproductive capacity of worms [58]. As a consequence, Src kinase was proposed as novel target for the future development of antischistosomal drugs.

In summary, PZQ at IC_50_ caused a general reduction of protein phosphorylation in *S. mekongi*. PZQ also altered expression and phosphorylation of many proteins in key pathways of the parasites, including calcium homeostasis, worm antigens and oxidative stress. Moreover, the differentially expressed and phosphorylated proteins indicated that PZQ interferes with protein folding and proteolysis through the ERAD pathway. Furthermore, kinases responsible for phosphorylation of previously identified pathways were predicted and Src kinase was found to be the main enzyme influenced in the four pathways of interest. Our findings suggest a novel effect of PZQ as well as indicating possible drug targets, which could be used for the treatment of Mekong schistosomiasis in the future.

## 4. Materials and Methods

### 4.1. S. Mekongi Life Cycle Maintenance, Animal Husbandry and Worm Collection

The life cycle of *S. mekongi* was maintained using snail *Neotricula aperta* as an intermediate host and mice *Mus musculus* as definitive hosts, as described earlier [59]. The snails were primarily collected from Mekong River in northeastern Thailand and cultured at the Applied Malacology Laboratory, Department of Social and Environmental Medicine, Faculty of Tropical Medicine, Mahidol University, Bangkok, Thailand. Eight-week-old female ICR mice were purchased from the National Laboratory Animal Center, Mahidol University. Mice were percutaneously infected with 25–30 cercariae of *S. mekongi* and housed in the Animal Care Unit, Faculty of Tropical Medicine, Mahidol University. Adult worms were collected at 8 weeks post-infection by hepatic perfusion with sterile 0.85% saline solution and subsequently cultured in Roswell Park Memorial Institute (RPMI) medium (Hyclone, GE Healthcare, Little Chalfont, UK) with 10% fetal calf serum (Hyclone) in a humidified 5% CO_2_ incubator at 37 °C until PZQ treatment.

All experiments involving animals were performed according to the ethical principles and guidelines for the use of animals at the National Research Council of Thailand (NRCT). In addition, all procedures were conducted with the permission from the Faculty of Tropical Medicine Animal Care and Use Committee (FTM-ACUC), Mahidol University, with approval number FTM-ACUC 014/2016.

### 4.2. PZQ Treatment and Half Maximal Inhibitory Concentration (IC_50_) Assessment

PZQ (Tokyo Chemical Industry, Tokyo, Japan) was mixed with Dimethyl sulfoxide (DMSO) prior to diluted with RPMI medium to concentrations of 0, 20, 40, 60, 80 and 100 μg/mL and administered for an hour to 10 pairs of *S. mekongi* for each concentration (three biologic replicates for each concentration). Worm movement was used as the indicator for viability. Periodic movement of the worms was observed under a video microscope (Celestron, CA, USA) at 0, 5, 10, 15, 30 and 60 min and worms that did not move after a minute of observation were identified as dead. After 1 hour of PZQ treatment, all worms were collected and kept at −80 °C for further experiments. Data on worm viability were plotted and statistical significance at 0.05 was calculated with ANOVA using SPSS for Windows version 15.0 (SPSS, Inc., Chicago, IL, USA). The concentration that gave approximately 50% viability was identified as IC_50_.

### 4.3. Protein Extraction and Phosphoprotein Enrichment

Protein extraction was performed within a week after PZQ treatment, using the frozen extraction method and phosphoprotein enrichment was performed using the TALON PMAC Magnetic Phospho Enrichment Kit (Takara, Shiga, Japan). First, 10 pairs of untreated and 40 μg/mL-PZQ-treated worms were put into a mortar and homogenized in liquid nitrogen. After the nitrogen had completely evaporated, lysis buffer (provided with the kit) was added and incubated on ice for 10 min. Protein lysate was transferred into tubes and centrifuged at 5000 × *g* for 5 min at 4 °C. The supernatant was transferred to new tube and the protein concentration was measured using the bicinchoninic acid (BCA) assay (Thermo Fisher Scientific, Waltham, MA, USA).

Phosphoprotein enrichment was performed according to manufacturer’s protocol. Briefly, 250 μg protein was mixed with metal ion-coated magnetic beads and mixed on a rotary shaker for 90 min at 4 °C. The supernatant was discarded, and proteins bound to beads were eluted using 75 μL of the provided elution buffer. Successful enrichment was confirmed using 12% sodium dodecyl sulfate–polyacrylamide gel electrophoresis (SDS-PAGE) and Pro-Q diamond staining (Thermo Fisher Scientific). All experiments were performed in three biologic replicates.

### 4.4. Protein Separation and In-Gel Digestion

For proteomic analysis, 50 μg of sample was analyzed by SDS-PAGE and resulting protein bands were visualized using Coomassie Blue G staining. For phosphoproteomic analysis, 15 μL of eluted sample was analyzed by SDS-PAGE gel and protein bands were visualized using silver staining. Thereafter protein bands from each lane were cut into small pieces for further in-gel digestion.

Coomassie dye was removed by incubating gels with 25-mM ammonium bicarbonate buffer containing 50% acetonitrile. Silver stain was removed with 30-mM potassium ferricyanide and 156-mM sodium thiosulfate in a 1:1 ratio. Gel pieces were then reduced with 4-mM dithiothreitol (Sigma-Aldrich, St. Louis, MO, USA) in 50-mM ammonium bicarbonate buffer, further alkylated with 250-mM iodoacetamide (Sigma-Aldrich) and dehydrated with 100% acetonitrile. Proteins were digested overnight with 10 ng trypsin (Sigma-Aldrich) dissolved in 200 μL of 50-mM ammonium bicarbonate buffer containing 5% acetonitrile. After digestion, peptides were extracted by adding 200 μL of acetonitrile. The supernatant containing eluted peptides was transferred to a new tube and dried using a speed vacuum, before being dissolved with 0.1% formic acid.

### 4.5. Protein Identification by MS

Peptide samples were analyzed using an UltiMate 3000 nano-liquid chromatography system (Thermo Fisher Scientific) coupled with a micrOTOF-Q electrospray ionization quadrupole time-of-flight MS (Bruker Daltonics, Billerica, MA, USA). Mobile phase A for sample injection was 2% acetonitrile and 0.1% formic acid in water and mobile phase B was 0.1% formic acid in acetonitrile with flow rate of 300 nl/min for 60 min. Data acquisition was performed using Hystar software (Bruker Daltonics). Spectra of the peptide covered mass ranges of *m/z* 400–3000 and 50–1500.

### 4.6. Data Analysis and Pathway Enrichment

Data from all gel pieces were merged into single Mascot generic file (.mgf) using MASCOT DEMON (Matrix Science, London, UK), which was then searched for *S. mekongi* proteins using an in-house transcriptome database with missed cleavages allowed [59]. Peptide tolerance was assigned to 200 ppm and the tandem MS tolerance was assigned to 0.6 Da. Methionine oxidation, cysteine carbamidomethylation, serine phosphorylation, tyrosine phosphorylation and threonine phosphorylation were identified as variable modifications. Proteins that showed differential expression or phosphorylation of more than 2-fold were selected for further pathway enrichment using BLAST2GO and KEGG Automatic Annotation Server (KAAS) applications.

BLAST2GO Version 5.2.3 (BioBam Bioinformatics, Valencia, Spain) was used to annotate sequences of differentially expressed or phosphorylated proteins according to their biologic processes, molecular function and subcellular localization with standard parameters [60]. Furthermore, protein sequences were analyzed and mapped into the KEGG Pathway using KEGG Automatic Annotation Server (KAAS: https://www.genome.jp/kegg/kaas/) with standard parameters [61]. The top 20 annotated pathways were shown, and pathways related to the effects of PZQ were presented with identified proteins highlighted.

### 4.7. Phosphorylation Level Investigation by Immunogold Labeling and Transmission Electron Microscope (TEM)

Phosphorylation levels of untreated and 40 μg/mL-PZQ-treated *S. mekongi* were assessed by phosphoserine immunogold labeling and TEM. Briefly, worms with and without treatment were fixed using 2.5% glutaraldehyde in 0.1 M sucrose phosphate buffer (SPB), pH 7.4, for an hour and subsequently washed 3 times with SPB for 10 min. Worm samples were fixed with secondary fixative 1% osmium tetroxide and dehydrated in graded ethanol. Samples were immersed in a series of LR white resin (Electron Microscopy Sciences, PA, USA) before embedding in pure LR white (Electron Microscopy Sciences) and polymerizing at 60 °C for 48 h. Sample blocks were cut into 100-nm-thick sections for further immunogold labeling.

Worm sections were initially blocked with 50-mM glycine in phosphate-buffered saline (PBS) and then with 5% bovine serum albumin (BSA) (Electron Microscopy Sciences) in PBS for 30 min each. Sections were incubated with primary antibody against phosphoserine (Merck, Darnstadt, Germany) for 1 hour, followed by washing three times with 0.1% BSA in PBS. Sections were incubated with secondary antibody conjugated with 10-nm gold particles (Electron Microscopy Sciences). After washing three times, a silver enhancement kit (Electron Microscopy Sciences) was used to improve the contrast of gold particle labeling. Finally, sections were stained with lead citrate and uranyl acetate prior to visualization in a HT7700 model TEM (Hitachi, Tokyo, Japan). The labeled gold particles were examined throughout the parasite body, especially on tegument, muscle and internal cells.

### 4.8. Kinase Prediction for Identified Pathways

**Figure 8:**
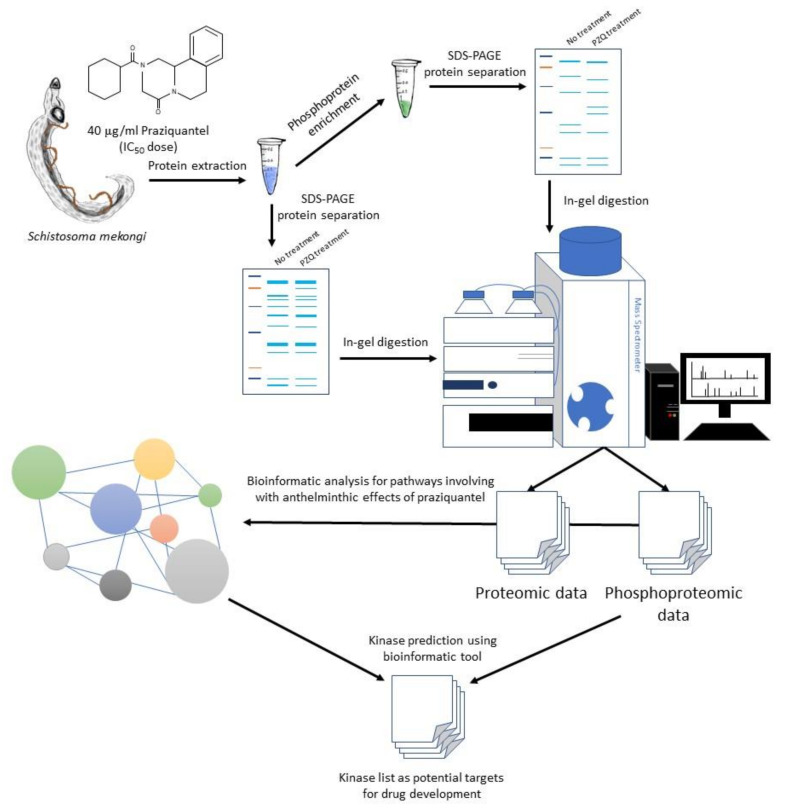
Graphical summary of the study. *S. mekongi* parasites were treated with IC_50_ dose of PZQ, anthelminthic drug. Protein lysate was extracted from untreated and PZQ-treated worms and subsequently enriched for phosphoproteins. Proteins and phosphoproteins were separated with 12% sodium dodecyl sulfate–polyacrylamide gel electrophoresis (SDS-PAGE) and protein bands were cut and digested by trypsin. Peptide was subjected to mass spectrometer for protein identification. Bioinformatics tools were used to analyze information from mass spectrometric data. Potential pathways involving with PZQ effects and kinases relating to those pathways were reported as possible targets for drug development.

Kinases that participate in calcium homeostasis, worm antigen, oxidative stress, protein folding and proteolysis pathways were predicted using the bioinformatic tool Group-based Prediction System version 5.0 (http://gps.biocuckoo.cn/online.php). The phosphopeptide data were input and analyzed with standard settings [62]. Predicted kinases were categorized according to their group and the number and percentage of phosphorylation sites of each predicted kinase were calculated. The graphical summary of whole experiments in this study is presented in Figure 8.

## Figures and Tables

**Table 1:** Top-20 *S. mekongi* proteins upregulated after 40 μg/mL-PZQ treatment.

No.	Accession No. Uniprot	Protein Name	MW	pI	Protein Score	Sequence Coverage	Average Fold-Change
**Structural protein**							
1	Q9Y1U7	Myosin light chain	18.3	4.5	37	31.3	2
2	Q26507	Paramyosin, partial	51.6	5.03	522	46	5.96
3	Q26595	Alpha tubulin	49.9	4.97	104	17.5	- ^1^
**Energy**							
4	A0A094ZC89	L-lactate dehydrogenase A chain	32.7	6.75	97	19.6	2
5	G4VP51	Putative ADP,ATP carrier protein	29.9	9.47	518	48.2	2.29
6	C1LV81	Aldolase	39.5	6.56	447	53.4	3.27
7	A0A095AJN4	Enolase	46.6	6.34	130	27.9	2.56
**Protease**							
8	C1L5C5	Putative aminopeptidase W07G4.4	56.2	7.14	360	25.7	2.31
9	A0A094ZYF3	Putative aminopeptidase W07G4.4	57.6	7.55	169	25.3	2.16
10	P43157	Cathepsin B	38.7	7.14	522	30.4	2.33
11	A0A095B296	Cathepsin B-like cysteine proteinase	38.6	7.51	43	38.8	2.05
**Antioxidant**							
12	Q26513	Glutathione-S-transferase	23.8	6.72	76	19.4	- ^1^
**Transcription and translation**							
13	C1LWP5	Eukaryotic translation elongation factor 1 alpha 2	25.7	7.67	149	36.5	3.69
**Immune system**							
14	C1L7Y4	Annexin A13 (Annexin XIII)	39.5	5.1	242	24.9	2.48
15	Q86DV3	Annexin	36.7	6.13	225	36.4	3.05
16	C1LKB8	Prohibitin-2 (B-cell receptor-associated protein BAP37)	28.8	9.72	226	38.5	3.29
**Antigen**							
17		55kD antigen	45.6	4.59	40	20.2	- ^1^
**Unknown**							
18	Q5DE25	SJCHGC06488 protein	30.2	5.54	232	25.9	3.54
19	Q5DGY1	Unknown	38.7	7.86	123	21.3	3.22
20	Q5DCK2	SJCHGC06304 protein	60.2	7.59	46	11.6	2.4

**^1^**protein only identified in PZQ treatment.

**Table 2:** Top 20 *S. mekongi* proteins downregulated after 40 μg/mL-PZQ treatment.

No.	Accession No.Uniprot	Protein Name	MW	pI	Protein Score	Sequence Coverage	AverageFold–Change
**Structural protein**							
1	G4VD36	Tropomyosin, partial	32.9	4.62	610	63.7	0 ^1^
2	Q02456	Myosin heavy chain	222.2	5.55	1003	27.8	0 ^1^
3	G4M0G1	Myosin heavy chain, putative	182.4	5.6	873	28.2	−2.0
**Energy**							
4	B2LXU1	Enolase	46.7	6.77	130	24.7	−3.12
5	C1LFP4	Putative aldehyde dehydrogenase 1B1 precursor	53.5	6.06	379	28.9	−4.0
6	G4LWI3	Aldehyde dehydrogenase, putative	53.7	5.76	113	19.8	−2.86
7	B2LXU3	Glyceraldehyde 3-phosphate dehydrogenase	36.5	7.68	152	33.4	−2.63
8	C7TRL1	Glyceraldehyde 3-phosphate dehydrogenase	36.5	8.4	340	33.7	−2.63
9	G4M130	Sm14 fatty acid-binding protein delta E3 variant	10.9	8.82	85	42.9	0^1^
**Stress response**							
10	G4VJ99	Putative heat shock protein	80.7	5.91	321	12.8	−2.27
**Signaling**							
11	A0A095B084	Pyruvate kinase PKM	62	6.63	136	19.3	−2.38
12	C1LEA0	TNF receptor-associated protein 1	80.2	6.24	321	9.8	−2.27
13	C1LFZ8	Arginine kinase	80.1	8.46	56	12.8	−2.27
14	A0A095ASB2	Inositol hexakisphosphate and diphosphoinositol–pentakisphosphate kinase 2	178	8.23	34	8.3	−2.0
**Unknown**							
15	Q5DAM7	SJCHGC06305 protein	61.1	6.48	278	28	−3.33
16	B3GUT7	Unknown	16	6.9	33	18.2	0 ^1^
17	Q5DBJ9	Unknown	38.8	8.44	69	22.2	−2.0
18	Q5C296	SJCHGC01885 protein, partial	111.8	5.26	924	37.3	−2.04
19	Q5DEG6	SJCHGC00214 protein	53.4	8.56	306	25.1	−2.22
20	Q5DGD4	SJCHGC06677 protein	90.6	5.08	61	12.5	−2.38

**^1^**protein only identified without treatment.

**Table 3:** Increased abundance of *S. mekongi* phosphoproteins after 40 μg/mL-PZQ treatment.

No.	Accession No. Uniprot	Protein Name	Phosphorylation Site		Average Fold–Change
			Peptide Sequence	Number	
**Structural protein**					
1	A5A6F8	Paramyosin	QASEDRATR	1 (ST)	2
			GVSPSTTRLESR	1 (ST)	
			MMNHDTESHVKISR	1 (ST)	
			TIYRGVSPSTTR	5 (ST), 1 (Y)	
			GMRATSMM	2 (ST)	
			ADMAERTVTVR	1 (ST)	
			AKSSLESQVDDLK	1 (ST)	
			LKAVSLEK	1 (ST)	
			GVSPSTTR	3 (ST)	
			QLTELNNAK	1 (ST)	
			LEGLDSQLNR	1 (ST)	
			LDEAGGSTTQTQELLKR	3 (ST)	
			ISRTIYR	2 (ST)	
			ATRLNNEVLR	1 (ST)	
			ELSVTSNRGMR	2 (ST)	
			KHAETELEEAQSR	1 (ST)	
2	Q05870	Paramyosin	GVSPSTTRLESR	1 (ST)	2.47
			MMNHDTESHVKISR	1 (ST)	
			TIYRGVSPSTTR	5 (ST), 1 (Y)	
			AERHAADLSYQVDALSER	1 (ST)	
			GMRATSMM	1 (ST)	
			ADMAERTVTVR	1 (ST)	
			AKSSLESQVDDLK	1 (ST)	
			VSELTIQVNTLSNDK	1 (ST)	
			LKAVSLEK	1 (ST)	
			GVSPSTTR	3 (ST)	
			QLTELNNAK	1 (ST)	
			LEGLDSQLNR	1 (ST)	
			LDEAGGSTTQTQELLKR	3 (ST)	
3	Q26507	Paramyosin, partial	ATRLNNEVLR	1 (ST)	- ^1^
4	A0A095BXK4	Actin-1	GTAASSSALEK	4 (ST)	- ^1^
			MQKEISALAPSTMK	1 (ST)	
			QEYDESGPSIVHRK	1 (ST), 1 (Y)	
			EISALAPSTMK	1 (ST)	
			DLYSNTVLSGGSTMYPGIADR	1 (ST), 1 (Y)	
5	A0A094ZID2	Actin-1	EICNLAPTTMKIK	2 (ST)	- ^1^
			KDLYSNIVLSGGSTMFPGISDR	2 (ST)	
**Energy**					
6	G4VBJ0	Putative malate dehydrogenase	RIQEAGTEVVEAK	1 (ST)	2.25
			AGAGSATLSMAYAGVR	2 (ST), 1 (Y)	
			FGISFRSFLTSSK	5 (ST)	
			LFGVTTLDVVR	1 (ST)	
			SFLTSSKHSPK	4 (ST)	
			AMICIITNPVNSTVPIAAEILK	2 (ST)	
7	A0A095CGL8	78 kDa glucose-regulated protein, partial	FDESTVQNDITHYPFSVVNKK	3 (ST), 1 (Y)	- ^1^
			DIKTNK	1 (ST)	
			EFAESYLGK	1 (ST)	
			FDESTVQNDITHYPFSVVNKK	3 (ST), 1 (Y)	
**Protease**					
8	P43157	Cathepsin B	DIMMYGPVEAAFDVYEDFLNYK	1 (Y)	- ^1^
			IAVYIVSLFTFLEAHVTTR	3 (ST)	
			MATLGTGMR	2 (ST)	
			AEISMLEGAVLDIRYGVSR	1 (ST), 1 (Y)	
			IDDEINTLMTGALENPNEEITATMDK	3 (ST)	
			YGVSRIAYSK	2 (ST), 1 (Y)	
			MATLGTGMRCLK	1 (ST)	
**Antigen**					
9	Q03528	22.6kd tegumental associated antigen	MATTEYRLSLMEQFIR	1 (ST)	- ^1^
			ATTEYR	1 (ST), 1 (Y)	
			ATTEYRLSLMEQFIR	1 (ST)	
10	P08418	70,000 mol wt antigen/hsp70 homologue (619 AA), partial	TTPSYVAFTDSER	3 (ST), 1 (Y)	- ^1^
			MFSPEEISSMVLTKMK	1 (ST)	
11	C1L7Y4	Annexin A13 (Annexin XIII)	IIGILGYR	1 (Y)	- ^1^
			MLLTDTDKMNAR	1 (ST)	
**Transcription and translation**					
12	G4VAD2	Putative elongation factor 1-alpha (EF-1-alpha)	TEDNPKCIK	1 (ST)	- ^1^
			ESGEMGKGSFK	1 (ST)	
			KESAAK	1 (ST)	
			MTDKPLR	1 (ST)	
			MDCTEPPFSEDR	1 (ST)	
13	A0A094ZN49	S phase cyclin A-associated protein in the endoplasmic reticulum	TASNTRSGSLISTNSR	2 (ST)	- ^1^
			SGSLISTNSR	2 (ST)	
14	G4M170	Meiotic checkpoint regulator cut4, putative	GISSSSR	2 (ST)	- ^1^
			HNSINMTTLR	1 (ST)	
			GASFSSSTFR	4 (ST)	
			CVDSLDSNKFLR	1 (ST)	
			YTGNIVSSECQNNVGHIFQSSKR	1 (ST)	
			GMLTFGILTGSK	1 (ST)	
			SSGSGLFYECSRK	3 (ST), 1 (Y)	
			WYIISNAPGTKSSYK	1 (ST)	
			CPDQTFTDSLYHAYGR	2 (ST)	
			VWASVIGRGMLTFGILTGSK	1 (ST)	
**Immune system**					
15	G4VL68	Putative annexin	STSRLVSR	2 (ST)	- ^1^
			CEVDMNTLKSMYR	1 (ST), 1 (Y)	
			GDSDTFIK	2 (ST)	
			EDTSGDFR	2 (ST)	
			CEVDMNTLK	1 (ST)	
			GLSTDEETITK	2 (ST)	
			NVVITVPAYFNDSQR	1 (ST)	
**Stress response**					
16	A0A094ZG26	Heat shock 70 kDa protein-like protein, partial	RTLSSST	4 (ST)	- ^1^
			LFSAEEISSMVLTK	1 (ST)	
17	Q8MXA4	Heat shock protein HSP60, partial	EEMEASNSEYEKEK	1 (ST), 1 (Y)	- ^1^
			DMAVGTGGIVFGDEADMYK	1 (ST), 1 (Y)	
			SAMLVGVDILADADAVTMGPKGR	1 (ST)	
18	O45038	HSP70	MKSSAESYLGK	2 (ST)	- ^1^
19	G4LWI2	Heat shock protein HSP60, putative	EEMEASNSEYEKEK	1 (ST), 1 (Y)	- ^1^
			VGNDGTITVKMK	1 (ST)	
**Transporter**					
20	A0A094ZQ18	Putative sodium-coupled neutral amino acid transporter 10	SFQPTLSNMR	3 (ST)	- ^1^
			NPPEPIDLTKIQSQMNVNNK	1 (ST)	
**Antioxidant**					
21	A0A095A0V3	Peroxiredoxin-2			- ^1^
22		Glutathione S-transferase	GIPGNSSMASK	2 (ST)	- ^1^
			AEISMLEGAVLDIRYGVSR	1 (ST), 1 (Y)	
			YGVSRIAYSK	2 (ST), 1 (Y)	
23	G4LXF8	26 Kd glutathione-S-transferase, Sm26, putative			- ^1^
**Unknown**					
24	Q5DBM4	SJCHGC01909 protein	MNHDSESHVK	2 (ST)	6.13
			GVSPSTTRLESR	1 (ST)	
			TIYRGVSPSTTR	5 (ST), 1 (Y)	
			AERHAADLSYQVDALSER	1 (ST)	
			GVSPSTTR	3 (ST)	
			QLTELNNAK	1 (ST)	
			LEGLDSQLNR	1 (ST)	
			MNHDSESHVK	2 (ST)	
			LDEAGGSTTQTQELLKR	3 (ST)	
			ISRTIYR	2 (ST)	
25	G4VM49	Hypothetical protein Smp_179350	WSSSSSNK	1 (ST)	- ^1^
			FSSTSSTR	3 (ST)	
			SINDNSSIEK	2 (ST)	
			TMRSLDSCIVPR	1 (ST)	
			ISGLTPVSTIFTGASNTTSTSTACGAVK	4 (ST)	
			LNTSR	1 (ST)	
			KQHSQSHDNLECSK	1 (ST)	
26	B3GUU7	Unknown			- ^1^
27	Q5DGY9	SJCHGC06312 protein	MAGESGTKTESQSDESTK	2 (ST)	- ^1^
			TESQSDESTK	1 (ST)	
			DSETAESIK	3 (ST)	
28	G4M072	Hypothetical protein Smp_153120	LSHSGVDDK	1 (ST)	- ^1^
			QCLNLTSYSK	1 (ST)	
			TPSSIKVYDLK	1 (ST)	
			SKYGWAPNSLLLGK	1 (Y)	
			MNDVTLSMVCNIR	1 (ST)	
			TSASNTVHTIHDITVKR	4 (ST)	

**^1^**phosphoprotein only identified in PZQ treatment.

**Table 4:** Decreased abundance of *S. mekongi* phosphoproteins after 40 μg/mL-PZQ treatment.

No.	Accession No. Uniprot	Protein Name	Phosphorylation Site		Average Fold–Change
			Peptide Sequence	Number	
**Structural protein**					
1	G4VLW1	Putative actin	GYSFTTTAER	2 (ST)	0 ^1^
			DSYVGDEAQSK	2 (ST)	
			GDEDVAALVIDNGSGMCK	1 (ST)	
			YSVWIGGSILASLSTFQQMWISK	1 (ST), 1 (Y)	
2	G4VD37	Putative tropomyosin	EKAEAEVAAMTR	1 (ST)	0 ^1^
			EESYEETIRDLTER	3 (ST), 1 (Y)	
			LLEEDLEVSSSRLTETLTK	2 (ST)	
**Energy**					
3	C1LV81	Aldolase	HNMVTAGQACK	1 (ST)	−2.38
			TLPTLLAERHIIPGIK	1 (ST)	
			TLPTLLAER	1 (ST)	
**Antigen**					
4	C1L9U2	Tegument antigen (I(H)A)	EGKVSTLPLVIQIIAATMSK	2 (ST)	0 ^1^
**Scaffold**					
5	G4VM27	Putative zinc finger protein	DNQSSNLEESFKHECDSDK	1 (ST)	0 ^1^
			YIQRSGLNR	1 (Y)	
			TFTTKSHLNK	2 (ST)	
			IKYSCVQCYK	1 (Y)	
			DNQSSNLEESFKHECDSDK	1 (ST)	
**Antioxidant**					
6	O97161	Thioredoxin peroxidase	KSGGLGHMK	1 (ST)	0 ^1^
**Transcription and translation**					
7	A0A094ZK92	Putative ATP-dependent RNA helicase DDX56	LSEVDGFRYR	1 (ST)	0 ^1^
			CRDVEYGVSR	1 (Y)	
			ELCSQVASNIKCLCK	1 (ST)	
8	G4VDN8	Putative dead box ATP-dependent RNA helicase	LSEVDGFRYR	1 (ST)	0 ^1^
			CRDVEYGVSR	1 (Y)	
			ELCSQVASNIKCLCK	1 (ST)	
**Stress response**					
9	A0A094ZZQ1	60 kDa heat shock protein, mitochondrial	SPKITK	1 (ST)	0 ^1^
			GTLAPAR	1 (ST)	
			GSKTDVDK	2 (ST)	
			DGVTVAKGIELK	1 (ST)	
			EESGANVAGMGGMGGMGGMGGMM	1 (ST)	
**Signaling**					
10	A0A095A3D3	20 kDa calcium-binding protein	FNNDSYK	1 (Y)	0 ^1^
			QCLTNHSNQSIR	1 (ST)	
			ENSSTMR	2 (ST)	
			SVRLSIIPLNPIYK	1 (ST), 1 (Y)	
11	G4LZ30	Calcium-binding protein, putative	LVSKENPSTMR	3 (ST)	0 ^1^
			SVRLSIIPLNPIYK	1 (ST), 1(Y)	
**Unknown**					
12	A0A095CG55	Hypothetical protein MS3_10497, partial	AQAVISQR	1 (ST)	0 ^1^
			SLLSALSTVYSHR	3 (ST), 1 (Y)	
13	G4VH32	Hypothetical protein Smp_179900	SQISNSEASNFDNSK	1 (ST)	0 ^1^
			VVVHSTVSMPFTPSGLSTGNR	2 (ST)	
14	C7TYF2	Hypothetical protein	TVINEMSLIPSINK	1 (ST)	0 ^1^
15	G4V5K0	Hypothetical protein Smp_057140	QSSSFFMR	1 (ST)	0 ^1^
			YIIASARQR	1 (ST)	
			SLLSALSTVYSHR	3 (ST), 1 (Y)	
			HSSGSDSVVGAITTR	4 (ST)	
			QSCSLQYKYIIASAR	1 (Y)	

**^1^**phosphoprotein only identified without treatment.

**Table 5:** Proteomic and phosphoproteomic analysis of proteins involved in calcium binding, worm antigen oxidative stress and protein folding and proteolysis pathway.

No.	Accession No. Uniprot	Protein Name	Status	
			Expression	Phosphorylation
**Proteins involving with calcium binding**				
1	C1L7Y4	Annexin A13 (Annexin XIII)	Up	–
2	C1LNX4	Voltage-dependent anion-selective channel protein 2	Down	–
3	G4M1U8	Voltage-dependent anion-selective channel, putative	Down	–
4	G4VL68	Putative annexin	Down	Up
5	Q03528	22.6kd tegumental associated antigen	–	Up
6	G4LZ30	Calcium-binding protein, putative	–	Down
7	A0A095A3D3	20 kDa calcium-binding protein	–	Down
8	C1L9U2	Tegument antigen (I(H)A)	–	Down
9	G4VH32	Hypothetical protein Smp_179900	–	Down
**Proteins involving with worm antigens**				
10	Q26595	Alpha tubulin	Up	–
11	Q86DV9	Similar to GenBank Accession Number Z29075 myophilin antigen in *Echinococcus granulosus*	Up	–
12	A0A094ZPJ2	Putative citrate synthase 2, mitochondrial	Up	–
13		55kD antigen	Up	–
14	Q06814	Antigen	Down	–
15	Q5D9C5	SJCHGC09453 protein	Down	–
16	P08418	70,000 mol wt antigen/hsp70 homolog (619 AA), partial	–	Up
17	Q03528	22.6kd tegumental associated antigen	–	Up
18	C1L9U2	Tegument antigen (I(H)A)	–	Down
19	A0A094ZZQ1	60 kDa heat shock protein, mitochondrial	–	Down
**Proteins involving with oxidative stress**				
20	C1LV40	Tryparedoxin peroxidase	Up	–
21	Q26513	Glutathione-S-transferase	Up	–
22	A0A094ZC89	L-lactate dehydrogenase A chain	Up	–
23	P37227	Malate dehydrogenase, partial	Up	–
24	C1LD38	Glyceraldehyde 3-phosphate dehydrogenase	Up	–
25	A0A095AJN4	Enolase	Up	–
26	Q7Z1I3	Lactate dehydrogenase-like protein	Up	–
27	A0A094ZPJ2	Putative citrate synthase 2, mitochondrial	Up	–
28	Q8WT66	Phosphoglycerate mutase	Up	–
29	C1LV81	Aldolase	Up	–
30	C1LFZ8	Arginine kinase	Down	–
31	A0A095B084	Pyruvate kinase PKM	Down	–
32	C7TRL1	Glyceraldehyde 3-phosphate dehydrogenase	Down	–
33	C1LD30	Glyceraldehyde 3-phosphate dehydrogenase	Down	–
34	B2LXU3	Glyceraldehyde 3-phosphate dehydrogenase	Down	–
35	B2LXU1	Enolase	Down	–
36	C1LFP4	Putative aldehyde dehydrogenase 1B1 precursor	Down	–
37	G4LWI3	Aldehyde dehydrogenase, putative	Down	–
38	A0A095A4B6	Aldehyde dehydrogenase X, mitochondrial	Down	–
39	Q5DAK2	SJCHGC06828 protein	Down	–
40	Q5BXP5	SJCHGC05927 protein, partial	Down	–
41	Q5DAM7	SJCHGC06305 protein	Down	–
42	Q5DFZ8	SJCHGC00411 protein	Down	–
43	C1L6T7	Hypothetical protein	Down	–
44	G4VBJ0	Putative malate dehydrogenase	–	Up
45	A0A095A0 V3	Peroxiredoxin-2	–	Up
46	G4LXF8	26 Kd glutathione-S-transferase, Sm26, putative	–	Up
47	O97161	Thioredoxin peroxidase	–	Down
**Proteins involving with protein folding and proteolysis**				
48	P43157	Cathepsin B	Up	Up
49	C1L5C5	Putative aminopeptidase W07G4.4	Up	–
50	A0A094ZYF3	Putative aminopeptidase W07G4.4	Up	–
51	A0A095B296	Cathepsin B-like cysteine proteinase	Up	–
52	C1LA34	Cathepsin B-like cysteine proteinase precursor	Up	–
53	C1L8N7	Putative Lysosomal Pro-X carboxypeptidase precursor	Up	–
54	G4LYP1	Mitochondrial processing peptidase beta-subunit (M16 family)	Up	–
55	G4VPS5	Putative chaperonin containing *t-*complex protein 1, theta subunit, tcpq	Up	–
56	A0A095AUU9	T-complex protein 1 subunit zeta	Up	–
57	A0A095CBQ7	T-complex protein 1 subunit theta	Up	–
58	A9CBJ4	DNA repair protein	Up	–
59	Q26551	Cyclophilin B	Up	–
60	Q5DC69	SJCHGC01960 protein, partial	Up	–
61	Q5DDG6	SJCHGC02536 protein	Up	–
62	Q5DE64	Unknown	Up	–
63	Q5C3A0	Unknown	Up	–
64	Q5DGY1	Unknown	Up	–
65	C1L6Z6	Mitochondrial processing peptidase	Down	–
66	C1LNT2	Serine protease inhibitor serpin	Down	–
67	O45039	HSP70, partial	Down	–
68	C7TZI9	Heat shock protein 90kDa alpha, partial	Down	–
69	G4VJ99	Putative heat shock protein	Down	–
70	C1LEA0	TNF receptor-associated protein 1	Down	–
71	Q06814	Calreticulin	Down	–
72	Q5BYY8	SJCHGC04813 protein, partial	Down	–
73	Q5C3 V3	SJCHGC05011 protein, partial	Down	–
74	Q5DBB0	SJCHGC02058 protein	Down	–
75	Q5DGD4	SJCHGC06677 protein	Down	–
76	Q5D9K8	Unknown	Down	–
77	Q5DBJ9	Unknown	Down	–
78	G4LWI2	Heat shock protein HSP60, putative	–	Up
79	Q8MXA4	Heat shock protein HSP60, partial	–	Up
80	Q5DGY9	SJCHGC06312 protein	–	Up
81	A0A094ZZQ1	60 kDa heat shock protein, mitochondrial	–	Down

## Supplementary Materials

The following are available online at https://www.mdpi.com/2076-0817/9/6/417/s1, Figure S1: Src kinase–substrate prediction from phosphopeptide data. Phosphopeptide data were used to predict kinases corresponding for their phosphorylation with Group-based Prediction System. Protein substrates of Src kinase are shown with number of phosphorylation sites and pathways. Src kinase was predicted to phosphorylate 85 sites of 14 proteins involved in calcium homeostasis, worm antigen, oxidative stress and proteins folding and proteolysis; Video S1: Movement of *S. mekongi* without PZQ treatment; Video S2: Movement of *S. mekongi* after treatment with 40 μg/mL of PZQ for 60 min; Table S1: *S. mekongi* proteins upregulated after 40 μg/mL-PZQ treatment; Table S2: *S. mekongi* proteins downregulated after 40 μg/mL-PZQ treatment; Table S3: Top 25 predicted kinases responsible for phosphorylation of proteins involved in calcium binding, worm antigen, oxidative stress, protein folding and proteolysis.
