# Supplementary material for: Effect of Praziquantel on Schistosoma mekongi Proteome and Phosphoproteome
Source: Pathogens. 2020 May 27;9(6):417. doi: 10.3390/pathogens9060417 (PMC7350297; doi:10.3390/pathogens9060417)
Supplement: Supplementary file 1 [file pathogens-09-00417-s001.zip › pathogens-793132 supplementary final/pathogens-793132 supplementary material .docx]

Effect of Praziquantel on *Schistosoma mekongi* Proteome and Phosphoproteome

Peerut Chienwichai ^1^, Sumate Ampawong ^2^, Poom Adisakwattana ^3^, Tipparat Thiangtrongjit ^4^, Yanin Limpanont ^5^, Phiraphol Chusongsang ^5^, Yupa Chusongsang ^5^ and Onrapak Reamtong ^4,^*

^1^ Faculty of Medicine and Public Health, HRH Princess Chulabhorn College of Medical Science, Chulabhorn Royal Academy, Bangkok 10210, Thailand; peerut.chi@pccms.ac.th

^2^ Department of Tropical Pathology, Faculty of Tropical Medicine, Mahidol University, Bangkok 10400, Thailand; sumate.aum@mahidol.ac.th

^3^ Department of Helminthology, Faculty of Tropical Medicine, Mahidol University, Bangkok 10400, Thailand; poom.adi@mahidol.ac.th

^4^ Department of Molecular Tropical Medicine and Genetics, Faculty of Tropical Medicine, Mahidol University, Bangkok 10400, Thailand; onrapak.rea@mahidol.ac.th

^5^ Department of Social and Environmental Medicine, Faculty of Tropical Medicine, Mahidol University, Bangkok 10400, Thailand; [yanin.lim@mahidol.ac.th](mailto:yanin.lim@mahidol.ac.th) (Y.L.); [phiraphol.chu@mahidol.ac.th](mailto:phiraphol.chu@mahidol.ac.th) (P.C.); [yupa.chu@mahidol.ac.th](mailto:yupa.chu@mahidol.ac.th) (Y.C.)

***** Correspondence: onrapak.rea@mahidol.ac.th


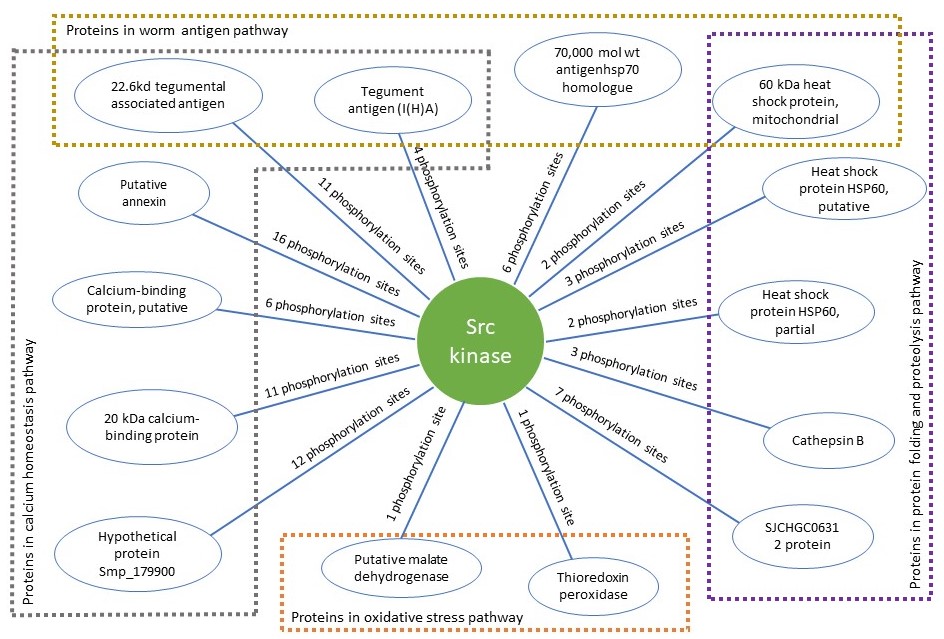


**Figure 1.** Src kinase–substrate prediction from phosphopeptide data. Phosphopeptide data were used to predict kinases corresponding for their phosphorylation with Group-based Prediction System. Protein substrates of Src kinase are shown with number of phosphorylation sites and pathways. Src kinase was predicted to phosphorylate 85 sites of 14 proteins involved in calcium homeostasis, worm antigen, oxidative stress and proteins folding and proteolysis.

**Table S1.** *S. mekongi* proteins up-regulated after 40 μg/ml PZQ treatment.

| Number | Accession No. IG | Accession No. Uniprot | Protein Name | MW | pI | Protein Score | Sequence Coverage | Average Fold-change |
| --- | --- | --- | --- | --- | --- | --- | --- | --- |
| 1 | gi\|5305329 | Q9Y1U7 | Myosin light chain | 18.3 | 4.5 | 37 | 31.3 | 2 |
| 2 | gi\|685936295 | A0A094ZC89 | L-lactate dehydrogenase A chain | 32.7 | 6.75 | 97 | 19.6 | 2 |
| 3 | gi\|685967348 | A0A095B296 | Cathepsin B-like cysteine proteinase | 38.6 | 7.51 | 43 | 38.8 | 2.05 |
| 4 | gi\|685965329 | A0A094ZYF3 | Putative aminopeptidase W07G4.4 | 57.6 | 7.55 | 169 | 25.3 | 2.16 |
| 5 | gi\|360043681 | G4VP51 | Putative ADP,ATP carrier protein | 29.9 | 9.47 | 518 | 48.2 | 2.29 |
| 6 | gi\|226468452 | C1L5C5 | Putative aminopeptidase W07G4.4 | 56.2 | 7.14 | 360 | 25.7 | 2.31 |
| 7 | gi\|11167 | P43157 | Cathepsin B | 38.7 | 7.14 | 522 | 30.4 | 2.33 |
| 8 | gi\|56756563 | Q5DCK2 | SJCHGC06304 protein | 60.2 | 7.59 | 46 | 11.6 | 2.4 |
| 9 | gi\|226471462 | C1L7Y4 | Annexin A13 (Annexin XIII) | 39.5 | 5.1 | 242 | 24.9 | 2.48 |
| 10 | gi\|685958231 | A0A095AJN4 | Enolase | 46.6 | 6.34 | 130 | 27.9 | 2.56 |
| 11 | gi\|29841472 | Q86DV3 | Similar to GenBank Accession Number AB063189 annexin B13a in Bombyx mori | 36.7 | 6.13 | 225 | 36.4 | 3.05 |
| 12 | gi\|56753443 | Q5DGY1 | Unknown | 38.7 | 7.86 | 123 | 21.3 | 3.22 |
| 13 | gi\|226479492 | C1LV81 | Aldolase | 39.5 | 6.56 | 447 | 53.4 | 3.27 |
| 14 | gi\|226485453 | C1LKB8 | Prohibitin-2 (B-cell receptor-associated protein BAP37) | 28.8 | 9.72 | 226 | 38.5 | 3.29 |
| 15 | gi\|56755505 | Q5DE25 | SJCHGC06488 protein | 30.2 | 5.54 | 232 | 25.9 | 3.54 |
| 16 | gi\|226481715 | C1LWP5 | Eukaryotic translation elongation factor 1 alpha 2 | 25.7 | 7.67 | 149 | 36.5 | 3.69 |
| 17 | gi\|487635 | Q26507 | Paramyosin, partial | 51.6 | 5.03 | 522 | 46 | 5.96 |
| 18 | gi\|1098303 |  | 55kD antigen | 45.6 | 4.59 | 40 | 20.2 | -^1^ |
| 19 | gi\|1389744 | Q26513 | Glutathione-S-transferase | 23.8 | 6.72 | 76 | 19.4 | -^1^ |
| 20 | gi\|161072 | Q26595 | Alpha tubulin | 49.9 | 4.97 | 104 | 17.5 | -^1^ |
| 21 | gi\|16904828 | Q8WT66 | Phosphoglycerate mutase | 28.2 | 7.01 | 33 | 26.8 | -^1^ |
| 22 | gi\|171473822 | Q5DC69 | SJCHGC01960 protein, partial | 12 | 5.88 | 134 | 49.5 | -^1^ |
| 23 | gi\|226469878 | C1LJ50 | Solute carrier family 2 | 56.9 | 8.45 | 25 | 7.5 | -^1^ |
| 24 | gi\|226470406 | C1L705 | SH3-domain GRB2-like endophilin B1 | 28 | 8.27 | 257 | 38.8 | -^1^ |
| 25 | gi\|226470520 | C1L762 | ATP synthase, H+ transporting, mitochondrial F1 complex, beta polypeptide | 29.9 | 5.6 | 409 | 55.3 | -^1^ |
| 26 | gi\|226472758 | C1L8N7 | Putative Lysosomal Pro-X carboxypeptidase precursor | 53.4 | 5.52 | 24 | 11.2 | -^1^ |
| 27 | gi\|226473754 | C1LA34 | Cathepsin B-like cysteine proteinase precursor | 37.2 | 8.62 | 69 | 19.1 | -^1^ |
| 28 | gi\|226474190 | C1LA53 | Ribosomal protein S10 | 17.8 | 10.05 | 57 | 22 | -^1^ |
| 29 | gi\|226477752 | C1LD38 | Glyceraldehyde 3-phosphate dehydrogenase | 36.4 | 8.6 | 382 | 37.3 | -^1^ |
| 30 | gi\|226477998 | C1LDB4 | Tropomyosin | 6.5 | 7.93 | 111 | 61.1 | -^1^ |
| 31 | gi\|226479410 | C1LV40 | Tryparedoxin peroxidase | 20.6 | 6.43 | 292 | 26.6 | -^1^ |
| 32 | gi\|29841466 | Q86DV9 | Similar to GenBank Accession Number Z29075 myophilin antigen in Echinococcus granulosus | 21.1 | 8.66 | 70 | 31.6 | -^1^ |
| 33 | gi\|31044498 | Q7Z1I3 | Lactate dehydrogenase-like protein | 36.1 | 8.65 | 96 | 13.9 | -^1^ |
| 34 | gi\|350645308 | G4LYP1 | Mitochondrial processing peptidase beta-subunit (M16 family) | 48.5 | 6.21 | 47 | 19.1 | -^1^ |
| 35 | gi\|350645626 | G4LXG7 | Leucine zipper protein, putative | 53.8 | 8.77 | 29 | 11.9 | -^1^ |
| 36 | gi\|350645943 | G4LWX4 | Ormdl proteins, putative | 79.9 | 8.96 | 68 | 8.7 | -^1^ |
| 37 | gi\|350646675 | G4LUW0 | Prohibitin, putative | 23.1 | 4.77 | 232 | 38.5 | -^1^ |
| 38 | gi\|353231491 | G4VDD4 | Hypothetical protein Smp_143570 | 225 | 8.39 | 27 | 8.9 | -^1^ |
| 39 | gi\|353231513 | G4VDJ9 | VPS13C protein, putative (fragment) | 398 | 6.3 | 21 | 4.8 | -^1^ |
| 40 | gi\|353231909 | G4VIL3 | Putative 40s ribosomal protein S10 | 17.7 | 10.05 | 57 | 17.6 | -^1^ |
| 41 | gi\|353232457 | G4VHE9 | Putative GTP-binding protein (I) alpha-2 subunit, gnai2 | 39.9 | 5.6 | 54 | 11 | -^1^ |
| 42 | gi\|353233111 | G4VLW1 | Putative actin | 41.7 | 5.3 | 3119 | 55.6 | -^1^ |
| 43 | gi\|360042651 | G4VGE0 | Putative replication factor A 1, rfa1 | 61.8 | 6.5 | 28 | 5.2 | -^1^ |
| 44 | gi\|360044422 | G4VTA4 | Putative tubulin beta chain | 48.8 | 4.97 | 216 | 24.3 | -^1^ |
| 45 | gi\|360044883 | G4VPS5 | Putative chaperonin containing t-complex protein 1, theta subunit, tcpq | 59.2 | 6.08 | 54 | 16 | -^1^ |
| 46 | gi\|552243 | P37227 | Malate dehydrogenase, partial | 14.9 | 6.2 | 199 | 43 | -^1^ |
| 47 | gi\|56752775 | Q5DHV7 | SJCHGC01881 protein | 29.1 | 4.26 | 111 | 23.6 | -^1^ |
| 48 | gi\|56753253 | Q5DH70 | SJCHGC01883 protein | 26.6 | 7.08 | 223 | 39.9 | -^1^ |
| 49 | gi\|56754309 | Q5DFR4 | SJCHGC02792 protein | 34 | 9.66 | 525 | 42.8 | -^1^ |
| 50 | gi\|56755425 | Q5DE64 | Unknown | 25.2 | 6.49 | 69 | 26.5 | -^1^ |
| 51 | gi\|56755924 | Q5DDG6 | SJCHGC02536 protein | 49.8 | 5.63 | 46 | 14.2 | -^1^ |
| 52 | gi\|56756751 | Q5DCA9 | SJCHGC06227 protein | 50.8 | 6.57 | 62 | 14.9 | -^1^ |
| 53 | gi\|56757207 | Q5DBN1 | SJCHGC00931 protein | 40 | 5.75 | 54 | 9.9 | -^1^ |
| 54 | gi\|56757229 | Q5DBM0 | SJCHGC00845 protein | 39.6 | 4.95 | 248 | 28 | -^1^ |
| 55 | gi\|60598652 | Q5C3A0 | Unknown | 21.8 | 8.52 | 52 | 19.5 | -^1^ |
| 56 | gi\|685956425 | A0A095AFH4 | Sodium/potassium-transporting ATPase subunit alpha | 95.7 | 5.99 | 77 | 12.2 | -^1^ |
| 57 | gi\|685956861 | A0A094ZJE5 | Dynein light chain 1, cytoplasmic | 10.1 | 6.9 | 47 | 32.2 | -^1^ |
| 58 | gi\|685957600 | A0A095BXC0 | Paramyosin | 81.5 | 5.42 | 1450 | 44 | -^1^ |
| 59 | gi\|685960373 | A0A094ZPJ2 | Putative citrate synthase 2, mitochondrial | 52 | 7.2 | 131 | 11.5 | -^1^ |
| 60 | gi\|685960923 | A0A094ZR23 | Sodium/potassium-transporting ATPase subunit alpha | 36.9 | 5.62 | 214 | 31.5 | -^1^ |
| 61 | gi\|685960938 | A0A094ZR35 | Hypothetical protein MS3_04935 | 43.8 | 9.11 | 60 | 7.7 | -^1^ |
| 62 | gi\|685961138 | A0A094ZRM9 | Mitochondrial import inner membrane translocase subunit Tim13-B, partial | 10.2 | 7.6 | 20 | 35.2 | -^1^ |
| 63 | gi\|685962802 | A0A094ZYC9 | Dolichyl-diphosphooligosaccharide--protein glycosyltransferase subunit 2 | 79.1 | 8.79 | 68 | 9.4 | -^1^ |
| 64 | gi\|685962981 | A0A095AUU9 | T-complex protein 1 subunit zeta | 46.9 | 6.33 | 77 | 13.9 | -^1^ |
| 65 | gi\|685963856 | A0A095AWE2 | Heterogeneous nuclear ribonucleoprotein K | 36.1 | 6.75 | 58 | 20.1 | -^1^ |
| 66 | gi\|685965261 | A0A095CBQ7 | T-complex protein 1 subunit theta | 55.6 | 5.48 | 54 | 14.5 | -^1^ |
| 67 | gi\|84657284 | A9CBJ4 | DNA repair protein | 38.6 | 4.53 | 46 | 17.5 | -^1^ |
| 68 | gi\|992633 | Q26551 | Cyclophilin B | 23.2 | 8.74 | 58 | 13.6 | -^1^ |

^1^ Proteins only identified after PZQ treatment.

**Table S2.** *S. mekongi* proteins down-regulated after 40 μg/ml PZQ treatment.

| Number | Accession  No. IG | Accession No. Uniprot | Protein Name | MW | pI | Protein Score | Sequence Coverage | Average Fold-change |
| --- | --- | --- | --- | --- | --- | --- | --- | --- |
| 1 | gi\|226480850 | C1LFP4 | Putative aldehyde dehydrogenase 1B1 precursor | 53.5 | 6.06 | 379 | 28.9 | 0.25 |
| 2 | gi\|56757978 | Q5DAM7 | SJCHGC06305 protein | 61.1 | 6.48 | 278 | 28 | 0.3 |
| 3 | gi\|186462279 | B2LXU1 | Enolase | 46.7 | 6.77 | 130 | 24.7 | 0.32 |
| 4 | gi\|350645988 | G4LWI3 | Aldehyde dehydrogenase,putative | 53.7 | 5.76 | 113 | 19.8 | 0.35 |
| 5 | gi\|186462283 | B2LXU3 | Glyceraldehyde 3-phosphate dehydrogenase | 36.5 | 7.68 | 152 | 33.4 | 0.38 |
| 6 | gi\|257209399 | C7TRL1 | Glyceraldehyde 3-phosphate dehydrogenase | 36.5 | 8.4 | 340 | 33.7 | 0.38 |
| 7 | gi\|56753850 | Q5DGD4 | SJCHGC06677 protein | 90.6 | 5.08 | 61 | 12.5 | 0.42 |
| 8 | gi\|685965992 | A0A095B084 | Pyruvate kinase PKM | 62 | 6.63 | 136 | 19.3 | 0.42 |
| 9 | gi\|226479066 | C1LEA0 | TNF receptor-associated protein 1 | 80.2 | 6.24 | 321 | 9.8 | 0.44 |
| 10 | gi\|226481457 | C1LFZ8 | Arginine kinase | 80.1 | 8.46 | 56 | 12.8 | 0.44 |
| 11 | gi\|353232145 | G4VJ99 | Putative heat shock protein | 80.7 | 5.91 | 321 | 12.8 | 0.44 |
| 12 | gi\|56755221 | Q5DEG6 | SJCHGC00214 protein | 53.4 | 8.56 | 306 | 25.1 | 0.45 |
| 13 | gi\|76154815 | Q5C296 | SJCHGC01885 protein, partial | 111.8 | 5.26 | 924 | 37.3 | 0.49 |
| 14 | gi\|350644665 | G4M0G1 | Myosin heavy chain, putative | 182.4 | 5.6 | 873 | 28.2 | 0.5 |
| 15 | gi\|56757271 | Q5DBJ9 | Unknown | 38.8 | 8.44 | 69 | 22.2 | 0.5 |
| 16 | gi\|685961709 | A0A095ASB2 | Inositol hexakisphosphate and diphosphoinositol-pentakisphosphate kinase 2 | 178 | 8.23 | 34 | 8.3 | 0.5 |
| 17 | gi\|161044 | Q02456 | Myosin heavy chain | 222.2 | 5.55 | 1003 | 27.8 | 0^1^ |
| 18 | gi\|161131 | G4VD36 | Tropomyosin, partial | 32.9 | 4.62 | 610 | 63.7 | 0^1^ |
| 19 | gi\|189502914 | B3GUT7 | Unknown | 16 | 6.9 | 33 | 18.2 | 0^1^ |
| 20 | gi\|20270936 | G4M130 | Sm14 fatty acid-binding protein delta E3 variant | 10.9 | 8.82 | 85 | 42.9 | 0^1^ |
| 21 | gi\|208659500 | C4PYI6 | Aquaporin | 32.8 | 8.18 | 35 | 6.3 | 0^1^ |
| 22 | gi\|226468664 | C1LNT2 | Serine protease inhibitor serpin | 28.1 | 4.95 | 46 | 8.9 | 0^1^ |
| 23 | gi\|226468748 | C1LNX4 | Voltage-dependent anion-selective channel protein 2 | 31.2 | 9.38 | 45 | 12.8 | 0^1^ |
| 24 | gi\|226470270 | C1L6T7 | Hypotherical protein | 51.3 | 8.5 | 33 | 16.4 | 0^1^ |
| 25 | gi\|226470388 | C1L6Z6 | Mitochondrial processing peptidase | 58.5 | 6.84 | 27 | 19.8 | 0^1^ |
| 26 | gi\|226475808 | C1LBB6 | Tubulin beta-2C chain | 23.4 | 5.32 | 149 | 49.1 | 0^1^ |
| 27 | gi\|226477782 | C1LD30 | Glyceraldehyde 3-phosphate dehydrogenase | 36.5 | 8.11 | 240 | 33.4 | 0^1^ |
| 28 | gi\|226478770 | C1LDV2 | UBX domain-containing protein 1 | 54.8 | 6.82 | 29 | 4.1 | 0^1^ |
| 29 | gi\|257215742 | C7TZI9 | Heat shock protein 90kDa alpha, partial | 29.2 | 4.42 | 178 | 33.8 | 0^1^ |
| 30 | gi\|2829291 | O45039 | HSP70, partial | 55.9 | 5.38 | 133 | 21.7 | 0^1^ |
| 31 | gi\|29840943 | Q86FD1 | Hypothetical protein, partial | 31.4 | 9.22 | 62 | 21.9 | 0^1^ |
| 32 | gi\|29841129 | Q86EU5 | Similar to GenBank Accession Number AF103726 peptide elongation factor 1-beta in Gallus gallus | 23.9 | 4.62 | 53 | 21.7 | 0^1^ |
| 33 | gi\|29841300 | Q86EC5 | Similar to GenBank Accession Number X72727 transformation upregulated nuclear protein in Homo sapiens | 29.6 | 9.22 | 38 | 23.4 | 0^1^ |
| 34 | gi\|29841388 | Q86E37 | Similar to GenBank Accession Number M80214 alpha tubulin in Schistosoma mansoni | 36.1 | 5.47 | 66 | 20.5 | 0^1^ |
| 35 | gi\|312018 | Q26593 | Protein disulfide isomerase homologue | 54.1 | 4.92 | 105 | 14.1 | 0^1^ |
| 36 | gi\|350644142 | G4M1U8 | Voltage-dependent anion-selective channel,putative | 31.3 | 9.21 | 48 | 20.9 | 0^1^ |
| 37 | gi\|350644668 | G4M0G4 | Myosin heavy chain, putative | 222 | 5.65 | 1262 | 30.8 | 0^1^ |
| 38 | gi\|350645610 | G4LXV1 | Rab-18, putative | 22.6 | 8.4 | 37 | 13.7 | 0^1^ |
| 39 | gi\|353231758 | G4VI62 | Putative annexin | 36.8 | 5.96 | 42 | 19.6 | 0^1^ |
| 40 | gi\|353232209 | G4VJG3 | Putative crp1/csrp1/crip1 | 19.9 | 8.9 | 28 | 29.6 | 0^1^ |
| 41 | gi\|353232400 | G4VH92 | Putative dro/myosuppressin receptor | 49.9 | 8.97 | 23 | 10.2 | 0^1^ |
| 42 | gi\|353233508 | G4VL68 | Putative annexin | 39.3 | 7.11 | 131 | 34.4 | 0^1^ |
| 43 | gi\|353233759 | G4VN76 | Putative tropomyosin | 27.2 | 4.49 | 333 | 43.5 | 0^1^ |
| 44 | gi\|360044360 | G4VSY1 | Putative p30 dbc protein | 100.1 | 5.82 | 43 | 6.7 | 0^1^ |
| 45 | gi\|360044806 | G4VPD0 | Putative skd/vacuolar sorting | 48.6 | 7.1 | 43 | 5.1 | 0^1^ |
| 46 | gi\|360044859 | G4VPQ1 | Putative histidine acid phosphatase | 69.2 | 8.22 | 34 | 13.1 | 0^1^ |
| 47 | gi\|3892185 | O96460 | Protein disulfide isomerase, partial | 54.1 | 5.9 | 55 | 14.6 | 0^1^ |
| 48 | gi\|407041 | Q26579 | Glucose transport protein | 56.7 | 8.6 | 45 | 5 | 0^1^ |
| 49 | gi\|499349 | Q06814 | Calreticulin | 42.9 | 4.73 | 40 | 12.9 | 0^1^ |
| 50 | gi\|552239 | Q06814 | Antigen | 45.3 | 4.69 | 33 | 14 | 0^1^ |
| 51 | gi\|56753623 | Q5DGP2 | SJCHGC02827 protein | 15.3 | 6.39 | 33 | 18.4 | 0^1^ |
| 52 | gi\|56754138 | Q5DFZ8 | SJCHGC00411 protein | 39.5 | 6.52 | 498 | 49.3 | 0^1^ |
| 53 | gi\|56754377 | Q5DFN0 | SJCHGC04562 protein | 13.2 | 7.77 | 33 | 21.9 | 0^1^ |
| 54 | gi\|56754784 | Q5DF29 | SJCHGC04243 protein | 19.5 | 9.33 | 38 | 5.3 | 0^1^ |
| 55 | gi\|56756885 | Q5DC42 | SJCHGC09302 protein | 46.7 | 8.4 | 62 | 17.1 | 0^1^ |
| 56 | gi\|56757457 | Q5DBB0 | SJCHGC02058 protein | 18 | 5.81 | 132 | 60.6 | 0^1^ |
| 57 | gi\|56758028 | Q5DAK2 | SJCHGC06828 protein | 14.9 | 8.56 | 32 | 34.9 | 0^1^ |
| 58 | gi\|56758584 | Q5D9S4 | SJCHGC09424 protein | 55.6 | 5.85 | 652 | 34.8 | 0^1^ |
| 59 | gi\|56758716 | Q5D9K8 | Unknown | 38.6 | 8.43 | 51 | 24.6 | 0^1^ |
| 60 | gi\|56758882 | Q5D9C5 | SJCHGC09453 protein | 88.7 | 5.55 | 56 | 16.5 | 0^1^ |
| 61 | gi\|56759388 | Q5D8M2 | SJCHGC06661 protein | 36.1 | 5.92 | 38 | 15.4 | 0^1^ |
| 62 | gi\|685961201 | A0A094ZQ50 | Tubulin alpha-2/alpha-4 chain | 48.2 | 4.96 | 50 | 11.3 | 0^1^ |
| 63 | gi\|685962224 | A0A094ZSE4 | Putative ATP-dependent RNA helicase DDX20 | 94.9 | 6.81 | 36 | 7.2 | 0^1^ |
| 64 | gi\|685962792 | A0A094ZTM9 | Dysferlin | 234.1 | 5.59 | 55 | 9.8 | 0^1^ |
| 65 | gi\|685962837 | A0A094ZYG2 | Kinesin-like protein KIF6, partial | 30.9 | 8.69 | 45 | 7.8 | 0^1^ |
| 66 | gi\|685963902 | A0A094ZXA3 | Glutamine synthetase | 46.1 | 6.46 | 35 | 13.1 | 0^1^ |
| 67 | gi\|685965976 | A0A095A4B6 | Aldehyde dehydrogenase X, mitochondrial | 56 | 7.08 | 197 | 20.1 | 0^1^ |
| 68 | gi\|685967905 | A0A095A306 | Hypothetical protein MS3_10216, partial | 10.8 | 8.75 | 20 | 32.3 | 0^1^ |
| 69 | gi\|76154176 | Q5C3V3 | SJCHGC05011 protein, partial | 24.3 | 9.41 | 45 | 14 | 0^1^ |
| 70 | gi\|76155451 | Q5C0T6 | SJCHGC02883 protein, partial | 29.3 | 8.69 | 219 | 18.7 | 0^1^ |
| 71 | gi\|76156157 | Q5BYY8 | SJCHGC04813 protein, partial | 24.5 | 5.04 | 66 | 24.1 | 0^1^ |
| 72 | gi\|76156528 | Q5BY06 | SJCHGC00991 protein, partial | 50.4 | 6.67 | 147 | 33.7 | 0^1^ |
| 73 | gi\|76156667 | Q5BXP5 | SJCHGC05927 protein, partial | 50.9 | 8.03 | 32 | 11.3 | 0^1^ |
| 74 | gi\|76156738 | Q5BXJ4 | SJCHGC04591 protein, partial | 22.4 | 9.68 | 29 | 3.1 | 0^1^ |

^1^ Proteins only identified without treatment.

**Table S3.** Top 25 predicted kinases responsible for phosphorylation of proteins involved in calcium binding, worm antigen, oxidative stress, protein folding, and proteolysis.

| No. | Kinase | Kinase Group | Number of phosphorylation sites for predicted kinase | | | |
| --- | --- | --- | --- | --- | --- | --- |
|  |  |  | **Proteins involving with calcium binding** | **Proteins involving with worm antigen** | **Proteins involving with oxidative stress** | **Proteins involving with protein folding and proteolysis** |
| 1 | Src | Dual | 60 | 30 | 21 | 56 |
| 2 | CAMKL | CAMK | 59 | 29 | 20 | 51 |
| 3 | WEE | Other | 58 | 29 | 21 | 52 |
| 4 | PEK | Other | 54 | 27 | 20 | 44 |
| 5 | GRK | AGC | 53 | 26 | 20 | 45 |
| 6 | PKC | AGC | 53 | 26 | 20 | 45 |
| 7 | CK1 | CK1 | 53 | 26 | 19 | 41 |
| 8 | STE20 | STE | 53 | 26 | 20 | 45 |
| 9 | LISK | TKL | 53 | 26 | 20 | 45 |
| 10 | STKR | TKL | 53 | 26 | 20 | 45 |
| 11 | Alpha | Atypical | 53 | 26 | 20 | 45 |
| 12 | TLK | Other | 53 | 26 | 20 | 45 |
| 13 | TOPK | Other | 53 | 26 | 20 | 45 |
| 14 | TTK | Other | 53 | 26 | 20 | 45 |
| 15 | PLK | Other | 52 | 24 | 19 | 40 |
| 16 | Lmr | Dual | 52 | 26 | 20 | 43 |
| 17 | DMPK | AGC | 51 | 27 | 20 | 45 |
| 18 | DAPK | CAMK | 51 | 26 | 20 | 45 |
| 19 | Haspin | Other | 51 | 25 | 20 | 41 |
| 20 | LRRK | TKL | 50 | 24 | 20 | 42 |
| 21 | DYRK | CMGC | 48 | 25 | 19 | 40 |
| 22 | VRK | CK1 | 46 | 22 | 18 | 43 |
| 23 | PIKK | Atypical | 46 | 24 | 19 | 41 |
| 24 | CDK | CMGC | 45 | 22 | 16 | 32 |
| 25 | NKF2 | Other | 45 | 24 | 19 | 37 |
